# Altered Whole-Brain Structural Covariance of the Hippocampal Subfields in Subcortical Vascular Mild Cognitive Impairment and Amnestic Mild Cognitive Impairment Patients

**DOI:** 10.3389/fneur.2018.00342

**Published:** 2018-05-22

**Authors:** Xuetong Wang, Yang Yu, Weina Zhao, Qiongling Li, Xinwei Li, Shuyu Li, Changhao Yin, Ying Han

**Affiliations:** ^1^School of Biological Science and Medical Engineering, Beihang University, Beijing, China; ^2^Beijing Advanced Innovation Centre for Biomedical Engineering, Beihang University, Beijing, China; ^3^Center of Alzheimer’s Disease, Beijing Institute for Brain Disorders, Beijing, China; ^4^Department of Neurology, XuanWu Hospital, Capital Medical University, Beijing, China; ^5^Department of Neurology, Hongqi Hospital, Mudanjiang Medical University, Mudanjiang, China

**Keywords:** hippocampal subfields, amnestic mild cognitive impairment, subcortical vascular mild cognitive impairment, structural covariance networks, MRI

## Abstract

The hippocampus plays important roles in memory processing. However, the hippocampus is not a homogeneous structure, which consists of several subfields. The hippocampal subfields are differently affected by many neurodegenerative diseases, especially mild cognitive impairment (MCI). Amnestic mild cognitive impairment (aMCI) and subcortical vascular mild cognitive impairment (svMCI) are the two subtypes of MCI. aMCI is characterized by episodic memory loss, and svMCI is characterized by extensive white matter hyperintensities and multiple lacunar infarctions on magnetic resonance imaging. The primary cognitive impairment in svMCI is executive function, attention, and semantic memory. Some variations or disconnections within specific large-scale brain networks have been observed in aMCI and svMCI patients. The aim of this study was to investigate abnormalities in structural covariance networks (SCNs) between hippocampal subfields and the whole cerebral cortex in aMCI and svMCI patients, and whether these abnormalities are different between the two groups. Automated segmentation of hippocampal subfields was performed with FreeSurfer 5.3, and we selected five hippocampal subfields as the seeds of SCN analysis: CA1, CA2/3, CA4/dentate gyrus (DG), subiculum, and presubiculum. SCNs were constructed based on these hippocampal subfield seeds for each group. Significant correlations between hippocampal subfields, fusiform gyrus (FFG), and entorhinal cortex (ERC) in gray matter volume were found in each group. We also compared the differences in the strength of structural covariance between any two groups. In the aMCI group, compared to the normal controls (NC) group, we observed an increased association between the left CA1/CA4/DG/subiculum and the left temporal pole. Additionally, the hippocampal subfields (bilateral CA1, left CA2/3) significantly covaried with the orbitofrontal cortex in the svMCI group compared to the NC group. In the aMCI group compared to the svMCI group, we observed decreased association between hippocampal subfields and the right FFG, while we also observed an increased association between the bilateral subiculum/presubiculum and bilateral ERC. These findings provide new evidence that there is altered whole-brain structural covariance of the hippocampal subfields in svMCI and aMCI patients and provide insights to the pathological mechanisms of different MCI subtypes.

## Introduction

The hippocampus is part of the limbic system. It plays important roles in memory processing, especially spatial memory (1). Studies have shown that the hippocampus can be affected by a variety of neurological diseases such as epilepsy and schizophrenia (2, 3). Importantly, hippocampal disruption is an early sign of Alzheimer’s disease (AD) and other forms of dementia (4).

However, the hippocampus is not a homogeneous structure, which consists of several subfields, specifically the cornu ammonis (CA) areas 1–4, the dentate gyrus (DG), the subiculum, and the presubiculum (5). The hippocampus subfields have distinct anatomy and functions (6). Notably, evidence supports the distinct connectivity between hippocampal subfields and other brain regions. The major input to the hippocampus is the performant path, coming from the entorhinal cortex (ERC) that connects with the DG and CA3 pyramidal neurons. In addition, the efferent fibers, which may originate from CA or subiculum, terminate in many brain regions (e.g., entorhinal area, posterior cingulate, medial frontal cortex, and gyrus rectus) (7). Previous studies reported that the hippocampal subfields were differently affected by many neurodegenerative diseases, especially mild cognitive impairment (MCI) (8, 9).

Mild cognitive impairment is a diagnosis given to older adults who have cognitive impairments but that does not interfere significantly with their daily activities (10). It is regarded as the transitional stage between normal aging and dementia. Amnestic mild cognitive impairment (aMCI) and subcortical vascular mild cognitive impairment (svMCI) are two subtypes of MCI, both associated with deficits in multiple cognitive domains, with the same chief complaints in memory deficits, but the pathogenesis of aMCI and svMCI are different (11, 12). The aMCI is characterized by episodic memory loss (13) and represents the prodromal stage of AD (14, 15). The svMCI is regarded as a prodromal stage of subcortical vascular dementia, showing extensive white matter hyperintensities and multiple lacunar infarctions on magnetic resonance imaging (16). The cognitive impairment of svMCI is mainly manifested in executive function, attention, and semantic memory (17–19). Importantly, some variations or disconnections within specific large-scale brain networks were observed in aMCI and svMCI patients (20–24). For example, patients with aMCI showed a pattern of brain disconnection between the posterior cingulate cortex (PCC), the medial prefrontal cortex (PFC), and the rest of the brain (21). A few studies have reported that aMCI patients were characterized by aberrance in resting-state functional connectivity of specific hippocampal subregions (such as DG and subiculum) (25, 26). Additionally, svMCI patients presented extensive decreased functional connectivity density and functional amplitude of spontaneous low-frequency oscillations in the medial PFC (22). However, it is unknown whether aMCI and svMCI patients have abnormalities in structural connections between hippocampal subfields and the cerebral cortex and whether these abnormalities are different between aMCI and svMCI.

Structural covariance networks (SCNs), based on voxel-based morphometry (VBM), generate a map of correlation between the gray matter (GM) volume of a region of interest and the other regions (27, 28). SCNs are regarded as the potential tool to reflect developmental coordination or synchronized maturation between regions of the brain (29). In addition, SCN analysis has been successfully applied to obtain the abnormality in brain connectivity in some neuropsychiatric disorders (30–32). In this study, SCNs were employed to characterize the structural connections between hippocampal subfields and the cerebral cortex. We selected five hippocampal subfields using an automated segmentation method as seeds to build the SCNs in aMCI patients, svMCI patients, and normal controls (NC). Finally, we compared the differences in strength of structural covariance between groups.

## Materials and Methods

### Participants

Patients with svMCI and aMCI were recruited through the memory clinic of the neurology department of Xuanwu Hospital, Capital Medical University, Beijing, China. Two experienced neurologists diagnosed all patients using the Petersen criteria (33). Healthy controls were recruited from the local community through advertisements. Subjects were excluded if they had the following clinical characteristics: (i) depressive symptoms with a Hamilton Depression Rating Scale score > 24; (ii) non-MCI disease that cause cognitive impairments, such as psychiatric disease, systemic disease, or alcohol or drug abuse; (iii) factors that would make neuropsychological testing infeasible, such as visual abnormalities, severe aphasia, or motor disorders. Written informed consent was obtained from all participants. According to the diagnostic criteria and exclusion criteria, there were 29 svMCI patients, 33 aMCI patients, and 36 NC subjects included in this study. All participants received a standardized clinical evaluation protocol including a global cognitive functioning test [i.e., Mini Mental Status Examination (MMSE)] and other cognitive assessments (i.e., AVLT). Table 1 shows the detailed demographic characteristics of the participants. This study was approved by the medical research ethics committee and the institutional review board of Xuanwu Hospital, Capital Medical University, Beijing, China.

**Table 1:** Demographics of participants [mean ± SD (range)].

	NC (*n* = 36)	svMCI (*n* = 29)	aMCI (*n* = 33)
Gender (M/F)	16/20	11/18	13/20
Age (years)	62.5 ± 6.6 (46–76)	63 ± 8.7 (46–77)	66 ± 8.4 (51–80)
Years of education	9.9 ± 4.6 (0–17)	8.6 ± 3.7 (0–17)	10.8 ± 4.1 (0–18)
AVLT-immediate recall	8.8 ± 1.9 (5.3–13.7)	6.9 ± 1.9 (3.3–10.3)*	6.0 ± 1.5 (3.3–9)*
AVLT-delayed recall	9.39 ± 3.26 (0–15)	6.2 ± 3.0 (0–13)*	3.5 ± 3.0 (0–12)*
AVLT-recognition	11.17 ± 2.68 (3–15)	10.07 ± 2.4 (3–14)	7.1 ± 4.2 (3–14)
MMSE	27.3 ± 2.3 (21–30)	25.6 ± 3.4 (16–30)	24.9 ± 3.1 (17–30)*
MoCa	26.0 ± 3.5 (15–30)	19.9 ± 3.9 (13–26)*	19.7 ± 4.1 (11–26)*

*ANOVA was performed, followed by Bonferroni post hoc analysis*.

*P < 0.05 between NC and svMCI or aMCI.

*NC, normal controls; svMCI, vascular mild cognitive impairment; aMCI, amnestic mild cognitive impairment; AVLT, auditory verbal learning test; MMSE, Mini Mental Status Examination; MoCa, Montreal Cognitive Assessment*.

### Image Acquisition

Structural MR images were acquired using sagittal magnetization-prepared rapid gradient echo (MP-RAGE) three-dimensional T1-weighted imaging sequence on a 3.0 T Siemens scanner at Xuanwu Hospital, Capital Medical University. The image parameters included repetition time (TR) = 1,900 ms; echo time (TE) = 2.2 ms; inversion time = 900 ms; flip angle = 9°; field of view = 224 mm × 256 mm; matrix size = 448 × 512; 176 slices; and slice thickness = 1.0 mm.

### Segmentation of Hippocampal Subfields

**Figure 1:**
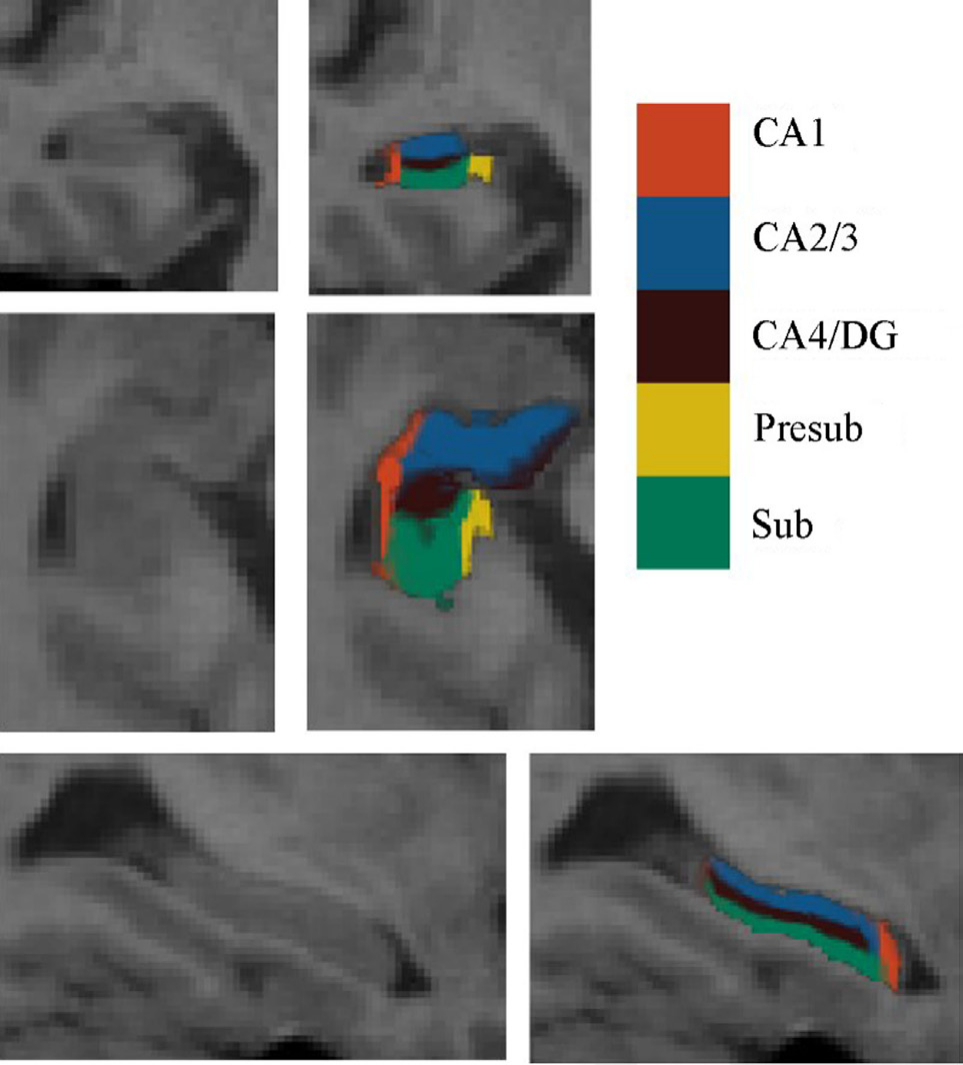
Right hippocampal subfield segmentation for one normal control subject. From left to right: cross-sectional slice of an MRI scan and corresponding automated segmentation of five subfields. Abbreviations: CA, cornu ammonis; DG, dentate gyrus; Presub, presubiculum; Sub, subiculum.

Automated segmentation of the hippocampal subfields was performed with the hippo-subfields module in FreeSurfer version 5.3,^1^ which uses the Bayesian statistical model built from manual segmentation of the right hippocampus in 0.38 mm × 0.38 mm × 0.8 mm *in vivo* MRI scans in 10 subjects (34). The results consisted of a collection of images that indicated each voxel’s posterior probability of belonging to different subregions in native space. By maximizing the posterior probability of the different subregions, the hippocampus of each subject was segmented to seven subfields: CA1, CA2/3, CA4/DG, presubiculum, subiculum, fimbria, and the hippocampal fissure. Previous research has reported that the fimbria and the hippocampal fissure showed relatively lower segmentation accuracies than other subfields (35, 36). Importantly, because the fimbria (white matter) and hippocampal fissure (cerebrospinal fluid) did not belong to GM, they were discarded in the subsequent SCN analysis. There is an illustration for the right hippocampal subfield segmentations for one NC subject in Figure 1.

### Image Processing

First, non-uniformity intensity correction of the structural magnetic resonance imaging data was performed with FreeSurfer. Then, the results after NU intensity correction were analyzed using Statistical Parametric Mapping software package in MATLAB (SPM12^2^). Following the inspection of image quality, we used VBM (VBM8 toolbox^3^) to extract the GM volume map of each subject (37). Additionally, we employed a spatially adaptive non-local denoising filter (38) and a hidden Markov random field model (39) to reduce the impact of noise in the GM volume map. Then, the images were transformed into the DARTEL template (40) from the Montreal Neurological Institute (MNI) space through the high-dimensional diffeomorphic anatomical registration using the exponentiated lie algebra (DARTEL) approach, which is a non-linear spatial normalization method. Subsequently, the voxel values were modulated to preserve regional volume information using the Jacobian determinants (41). Finally, we smoothed the modulated images using Gaussian Kernel specified in 12 mm full width at half maximum.

### Definition of Seed Regions

**Figure 2:**
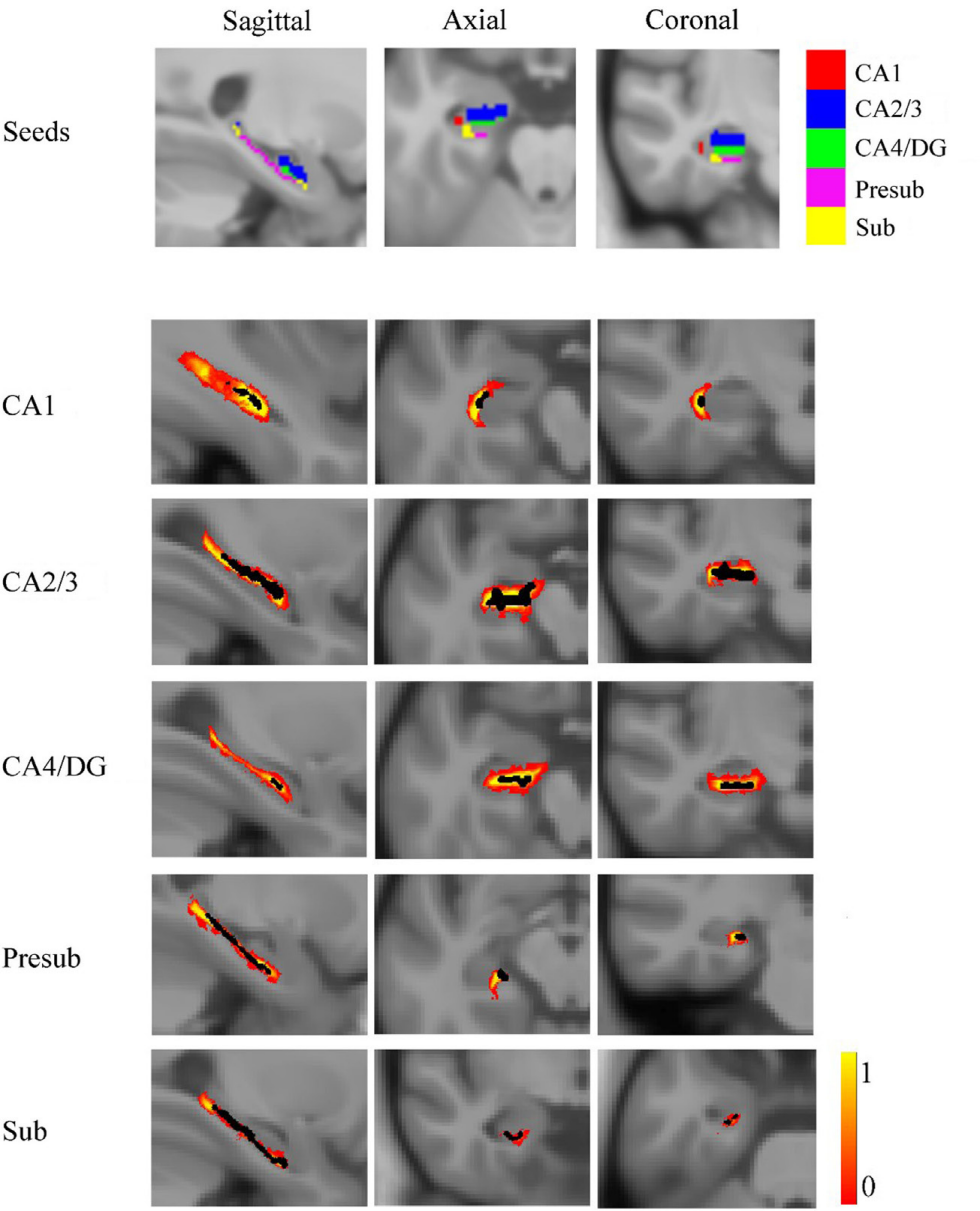
The seed regions compared to probabilistic atlases. At the top of the figure, the seeds are shown in Montreal Neurological Institute space. In the lower part of the figure, the probabilistic atlases are viewed in heat color maps, and the volumes of seeds are overlaid with black color. Abbreviations: CA, cornu ammonis; DG, dentate gyrus; Presub, presubiculum; Sub, subiculum.

For each subject, the deformation field derived from the NU intensity corrected image to normalized image was applied to the hippocampal subfields’ label image in native space. To reduce the possible impact of segmentation inaccuracy on subsequent analysis, the transformed hippocampal subfield labels were combined for all subjects and the 100% overlapped regions were selected. Then, these regions on each side were masked using the hippocampal label from the Harvard-Oxford subcortical structural atlas. Additionally, if there existed overlap for any two hippocampal subfields, the overlapped regions were removed. After that, the seed region for each hippocampal subfield was defined in MNI space. All the seed regions (in black color) were overlaid to the probabilistic atlas (in Heat color) of hippocampal subfields (34), as shown in Figure 2. The seeds almost located within the atlas.

### Construction of Structural Covariance Networks

For each group, the strength of structural covariance between each subfield seed and all other regions across the whole brain were obtained by applying multiple regression models in SPM12 to perform a voxel-based statistical analysis on the smoothed and modulated GM image. We imported the extracted mean GM volume from each seed as a covariate. As the age and gender would influence the GM volumes, we removed the effects of gender and age on the structural covariance networks by entering them as confounding covariates. The resulting covariance patterns were employed with thresholds at *P* < 0.05 with the false discovery rate (FDR) correction and reserved positive covariance. Finally, the results were displayed on the MNI template in the BrainNet Viewer software^4^ (42).

### Between-Group Differences in the Structural Association

Many studies have indicated that the different slopes for any pair of voxels may represent the difference in their structural association (43, 44). To evaluate the difference in strength of structural covariance between groups, we performed a between-group analysis of slopes. The analysis used a multiple classic interaction linear model:

$$y=\beta_{0}+\beta_{1}X+\beta_{2}G+\beta_{3}(G\times X)+\beta_{4}\text{Age}+\beta_{\text{5}}\text{Gender}+\varepsilon$$

*G* was used as a grouping variable, and two groups were put into the same model, where *G* = 1 for the one group, and *G* = 0 for another group. The gender and age may affect the association of two voxels, so they were considered as independent variables in a linear model, where *X* represented the averaged GM volume in each seed, and *y* represented the GM volumes of each voxel in whole brain. Then, the linear regression model between *y* and *X* was adjusted by adding a gender term *Gender*, an age term *Age*, a group term *G*, and an interaction term *G* × *X*. Specific *t*-value contrasts were established to map the significant different voxels in slopes between any two groups. The significant differences between groups were obtained based on the two-tailed Gaussian random field (GRF) correction, with a voxel level of *P* < 0.01 and a cluster level of *P* < 0.05.

## Results

### Demographics

Table 1 shows demographics of the healthy controls, svMCI patients, and aMCI patients. There were no significant differences in sex, age, and years of education between groups. However, significant differences between groups were found in the AVLT-immediate recall (*F* = 12.059, *P* < 0.001), AVLT-delayed recall (*F* = 11.501, *P* < 0.001), AVLT-recognition recall (*F* = 2.804, *P* = 0.066), MMSE (*F* = 3.3765, *P* = 0.27), and Montreal Cognitive Assessment (*F* = 27.276, *P* < 0.001) through one-way analysis of variance. The following *post hoc* test revealed that AVLT-immediate recall, AVLT-delayed recall, and MoCa in patients of aMCI and svMCI were significantly lower than scores in controls. In addition, the score of MMSE was significantly lower in the aMCI group than in the control group, but there was no significant difference in score of MMSE between the svMCI and NC groups.

### Structural Covariance Networks Within Groups

**Figure 3:**
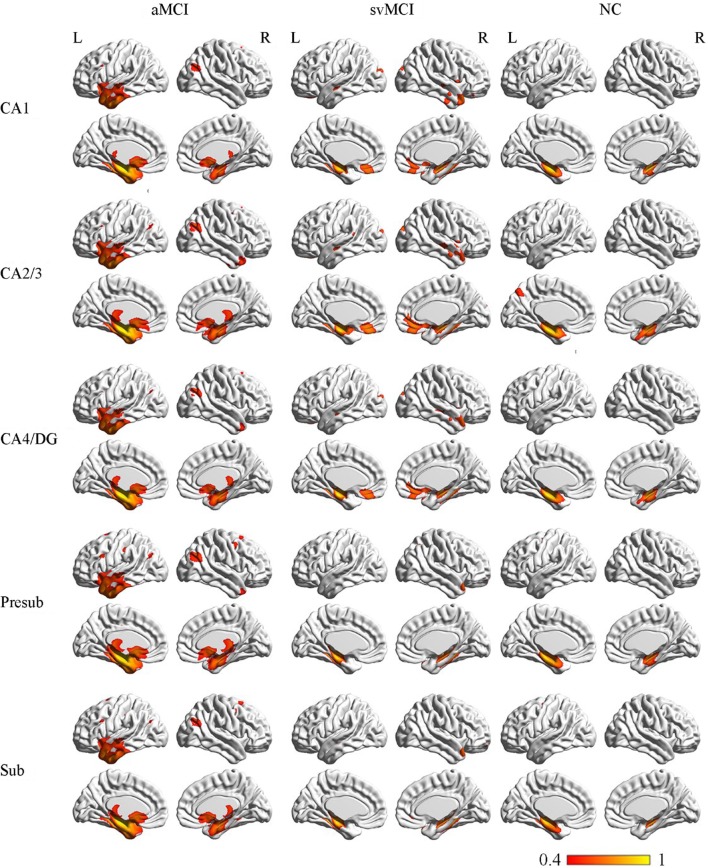
Structural covariance networks of left hippocampal subfields in the three groups. Statistical maps of regions significantly correlated with the seed region in each group. The results are presented as CC values (*P* < 0.05, false discovery rate corrected). Abbreviations: L, left; R, right; CC, correlation coefficient; NC, normal controls; svMCI, vascular mild cognitive impairment; aMCI, amnestic mild cognitive impairment; CA, cornu ammonis; DG, dentate gyrus; Presub, presubiculum; Sub, subiculum.

**Figure 4:**
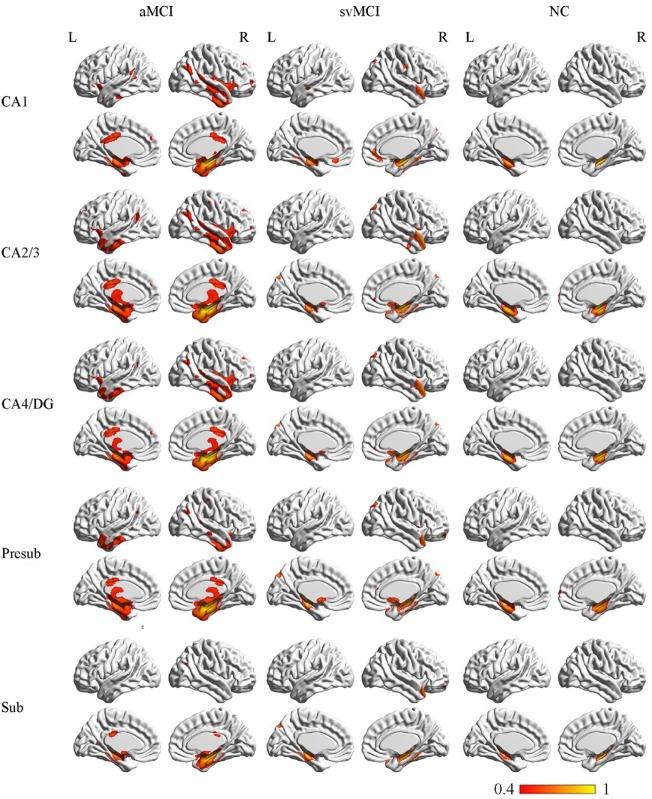
Structural covariance networks of right hippocampal subfields in the three groups. Statistical maps of regions significantly correlated with the seed region in each group. The results are presented as CC values (*P* < 0.05, false discovery rate corrected). Abbreviations: L, left; R, right; CC, correlation coefficient; NC, normal controls; svMCI, subcortical vascular mild cognitive impairment; aMCI, amnestic mild cognitive impairment; CA, cornu ammonis; DG, dentate gyrus; Presub, presubiculum; Sub, subiculum.

The SCN patterns of the left and right hippocampal subfields in the three groups are shown in Figures 3 and 4, respectively. Each of the hippocampal subfield seed regions covaried with the ERC and fusiform gyrus (FFG) among the three groups. The regions showing significant correlations with hippocampal subfields were relatively larger in the aMCI group than the svMCI group and NC group.

#### Structural Covariance Networks in the aMCI Group

##### Left

In the aMCI group, in addition to the ERC and FFG, the left CA1 covaried with the left temporal pole (TP), right angular gyrus, subcallosal cortex, and thalamus. For the left CA2/3 network, the structural maps involved the ERC and FFG, subcallosal cortex, thalamus, and angular gyrus. The left CA4/DG correlated regions were similar to the regions in the left CA2/3 network in the aMCI group, but it additionally included the right superior frontal gyrus. For the aMCI group, the left subiculum and left presubiculum covariance maps involved the left TP, subcallosal cortex, thalamus, superior and middle frontal gyrus, right middle occipital gyrus, and bilateral angular gyrus.

##### Right

In the aMCI group, the right CA1 covaried with the ERC and FFG, right TP, PCC, and angular gyrus. CA2/3 showed significant correlation with entorhinal areas, thalamus, bilateral TP, middle frontal gyrus, PCC, and angular gyrus. The CA4/DG correlated regions were similar to the regions covaried with the right CA2/3 subfield in the aMCI group. For the right presubiculum networks, the covariance maps of the right presubiculum involved entorhinal areas, thalamus, bilateral TP, PCC, and angular gyrus. The subiculum showed significant covariance with the ERC, FFG, and right angular gyrus.

#### Structural Covariance Networks in the svMCI Group

##### Left

The left CA1 showed significant correlations with the ERC, FFG, superior occipital gyrus, orbitofrontal cortex (OFC), and right TP in the svMCI group. The covariance maps of the left CA2/3 involved the FFG, right TP, OFC, occipital pole, and entorhinal areas in the svMCI group. The left CA4/DG covariance maps were similar to the covariance maps of the left CA2/3 in the svMCI group. In the svMCI group, the maps of the left presubiculum and left subiculum were virtually identical, and the covaried regions included the FFG, ERC, fusiform, and right TP.

##### Right

The right CA1 covaried with the ERC, FFG, superior occipital gyrus, right TP, and OFC in the svMCI group. The right CA2/3 covaried with entorhinal areas, right TP, and superior occipital gyrus. The right CA4/DG covaried with entorhinal areas, fusiform, right TP, superior occipital gyrus, and subcallosal cortex. The regions covaried with the right presubiculum were similar to those regions connected with the right CA4/DG subfield in the svMCI group. In addition, the covariance maps of the right subiculum hippocampal subfields involved entorhinal areas, fusiform, right TP, and superior occipital gyrus.

#### Structural Covariance Networks in the NC Group

##### Left

The left CA1 covaried with the bilateral ERC and FFG in the NC group. In addition to the ERC and FFG, the covariance regions with the left CA2/3 also included the left precuneus cortex. The left CA4/DG covariance maps were extremely similar to the maps of the left CA1 subfield in the NC group. For the left subiculum and left presubiculum networks, both covariance maps involved the left precentral gyrus, FFG, and ERC in NC subjects.

##### Right

The right CA1 covaried with the ERC and FFG in the NC group. In addition to the ERC and FFG, the covariance regions with the right CA2/3 also involved the right OFC. The right CA4/DG correlated regions included the ERC and FFG in the NC group. The right presubiculum showed significant correlations with the right OFC, FFG, and ERC in NC subjects. The right subiculum covaried with the ERC and FFG.

### Significant Difference in the Structural Associations Between Groups

#### aMCI Group vs. NC Group

**Figure 5:**
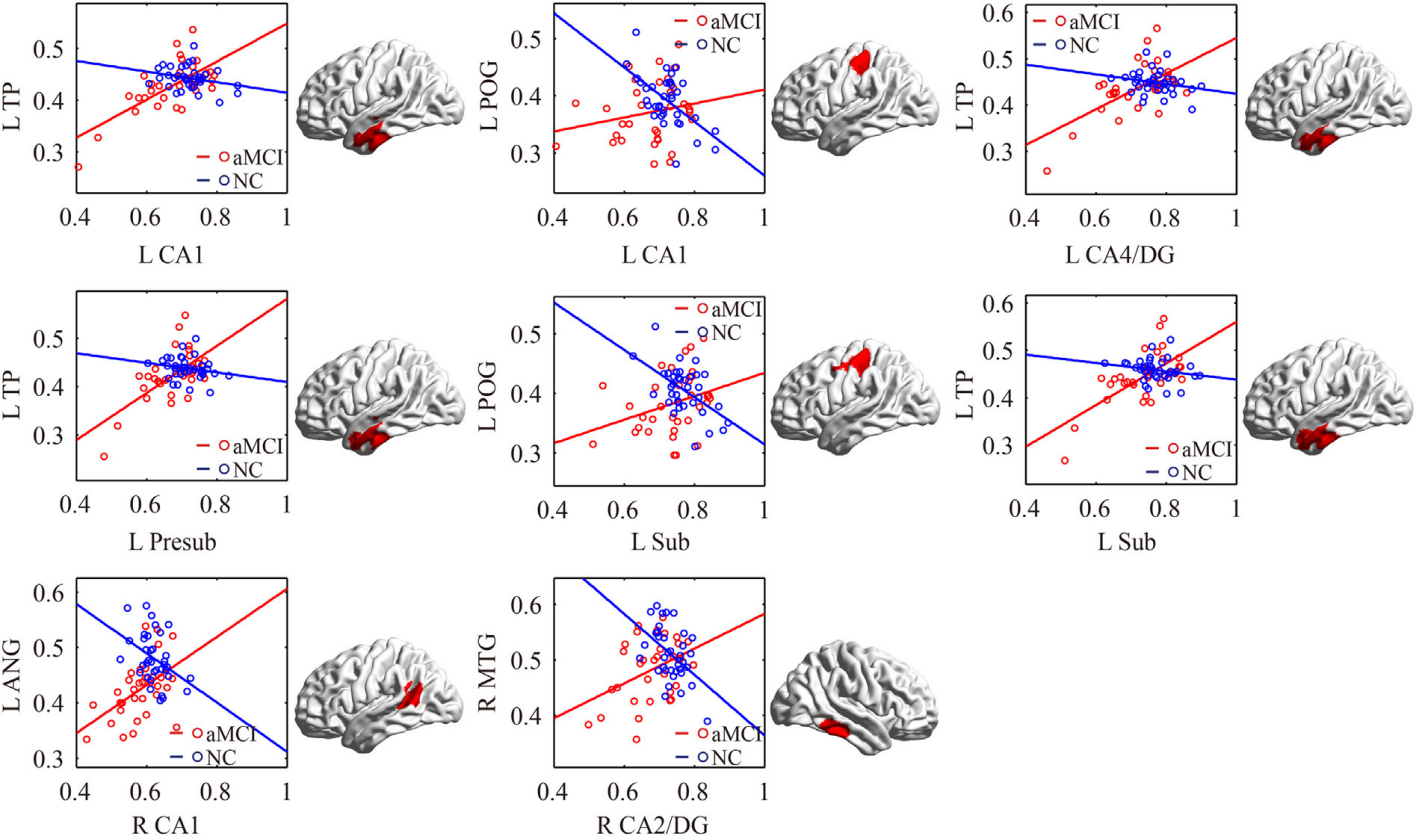
Significant between-group differences in structural association for aMCI and NC. A cluster showing significant structural difference (Gaussian random field-corrected at voxel level: *P* < 0.01 and cluster level: *P* < 0.05) between aMCI and NC is presented on the right, and a plot of slope differences between the seed region and cluster region is presented on the left. Abbreviations: L, left; R, right; NC, normal controls; aMCI, amnestic mild cognitive impairment; CA, cornu ammonis; DG, dentate gyrus; Presub, presubiculum; Sub, subiculum; TP, temporal pole; POG, postcentral gyrus; ANG, angular gyrus.

There were some significant differences observed between the aMCI group and NC group when the strength of the structural correlations was considered (Table 2; Figure 5). There was a significant increased association between the hippocampal subfields and other brain regions that was found in the aMCI group compared to the NC group. The left CA1, left CA4/DG, left presubiculum, and left subiculum showed increased covariance with the left pole in the aMCI group compared to NC. In addition, increased significant covariance was found between the left CA1/left subiculum and left postcentral gyrus (POG), right CA2/3 and right middle/inferior temporal gyrus (ITG), and right CA1 and left angular gyrus in the aMCI group compared to the NC group.

**Table 2:** Significant between-group differences in structural association between selected regions of interest and other cortical areas.

Contrast	Seed	BA	Region	MNI coordinates			Peak intensity	Cluster size (voxels)
				*X*	*Y*	*Z*		
**aMCI vs. NC**								
aMCI > NC	L_CA1	21/20	L TP	−60	6	−23	3.58	3,132
	L_CA1	3/4/6	L POG/PRG	−47	−23	63	3.75	1,472
	L_CA4/DG	21/20	L TP	−59	6	−23	3.49	2,047
	L_presubiculum	21/20	L TP	−57	9	−26	3.44	5,562
	L subiculum	3/4/6	L POG/PRG	−50	−23	60	3.93	1,796
	L subiculum	21/20	L TP	−57	8	−23	3.63	3,135
	R_CA1	40/39	L ANG/SMG	−51	−51	15	3.76	1,594
	R_CA2/3	21/20	R MTG/ITG	53	−33	−17	3.71	1,386

**svMCI vs. NC**								
svMCI > NC	L_CA1	11/10	OFC	−8	39	−23	4.23	2,359
	R_CA1	11/10	OFC	−8	39	−17	3.91	2,432
	R_CA1	10	R PFC	36	62	11	3.99	1,657
	L_CA2/3	11	OFC	−5	41	−24	3.91	1,564
	L_presubiculum	36/37	R FFG	35	−33	−14	4.05	2,261

**aMCI vs. svMCI**								
aMCI > svMCI	L_presubiculum	34/28/35	L ERC/PRC	−11	−6	−21	4.58	1,653
	L_subiculum	34/28/35	L ERC/PRC	−11	−5	−21	4.17	1,811
	R_prsubiculum	34/28/35	R ERC/PRC	15	−9	−21	4.37	1,188
	R_subiculum	34/28/35	R ERC/PRC	15	−9	−26	3.79	1,214

svMCI > aMCI	L_CA4/DG	36/37	R FFG	35	−33	−17	4.36	1,403
	L_presubiculum	36/37	R FFG	27	−33	−14	4.32	1,987
	L_subiculum	36/37	R FFG	38	−38	−11	4.59	2,208

*The regions listed showed significant between-group differences (Gaussian random field-corrected at voxel level: *P* < 0.01 and cluster level: *P* < 0.05), and peak coordinates are reported in standard MNI space*.

*BA, Brodmann area; L, left; R, right; NC, normal controls; svMCI, vascular mild cognitive impairment; aMCI, amnestic mild cognitive impairment; CA, cornu ammonis; DG, dentate gyrus; ERC, entorhinal cortex; FFG, fusiform gyrus; TP, temporal pole; MTG, middle temporal gyrus; ITG, inferior temporal gyrus; POG, postcentral gyrus; PRG, precentral gyrus; PRC, perirhinal cortex; OFC, orbitofrontal cortex; PFC, prefrontal cortex; SMG, supramarginal gyrus; ANG, angular gyrus*.

#### svMCI Group vs. NC Group

**Figure 6:**
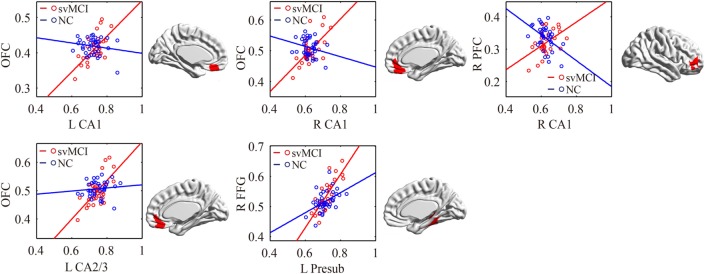
Significant between-group differences in structural association for svMCI and NC. A cluster showing significant structural difference (Gaussian random field-corrected at voxel level: *P* < 0.01 and cluster level: *P* < 0.05) between svMCI and NC is presented on the right, and a plot of slope differences between the seed region and cluster region is presented on the left. Abbreviations: L, left; R, right; NC, normal controls; svMCI, subcortical vascular mild cognitive impairment; CA, cornu ammonis; DG, dentate gyrus; Presub, presubiculum; Sub, subiculum; OFC, orbitofrontal cortex; PFC, prefrontal cortex; FFG, fusiform gyrus.

The significant differences of the association slope between the svMCI group and the NC group are shown in Table 2 and Figure 6. There were significant increased associations between the bilateral CA1/left CA2/3 and OFC in the svMCI group compared to the NC group. Then, the left presubiculum showed increased covariance with the right FFG in the aMCI group relative to the svMCI group. The right CA1 showed increased covariance with the right prefrontal gyrus in the svMCI group compared to the NC group.

#### aMCI Group vs. svMCI Group

**Figure 7:**
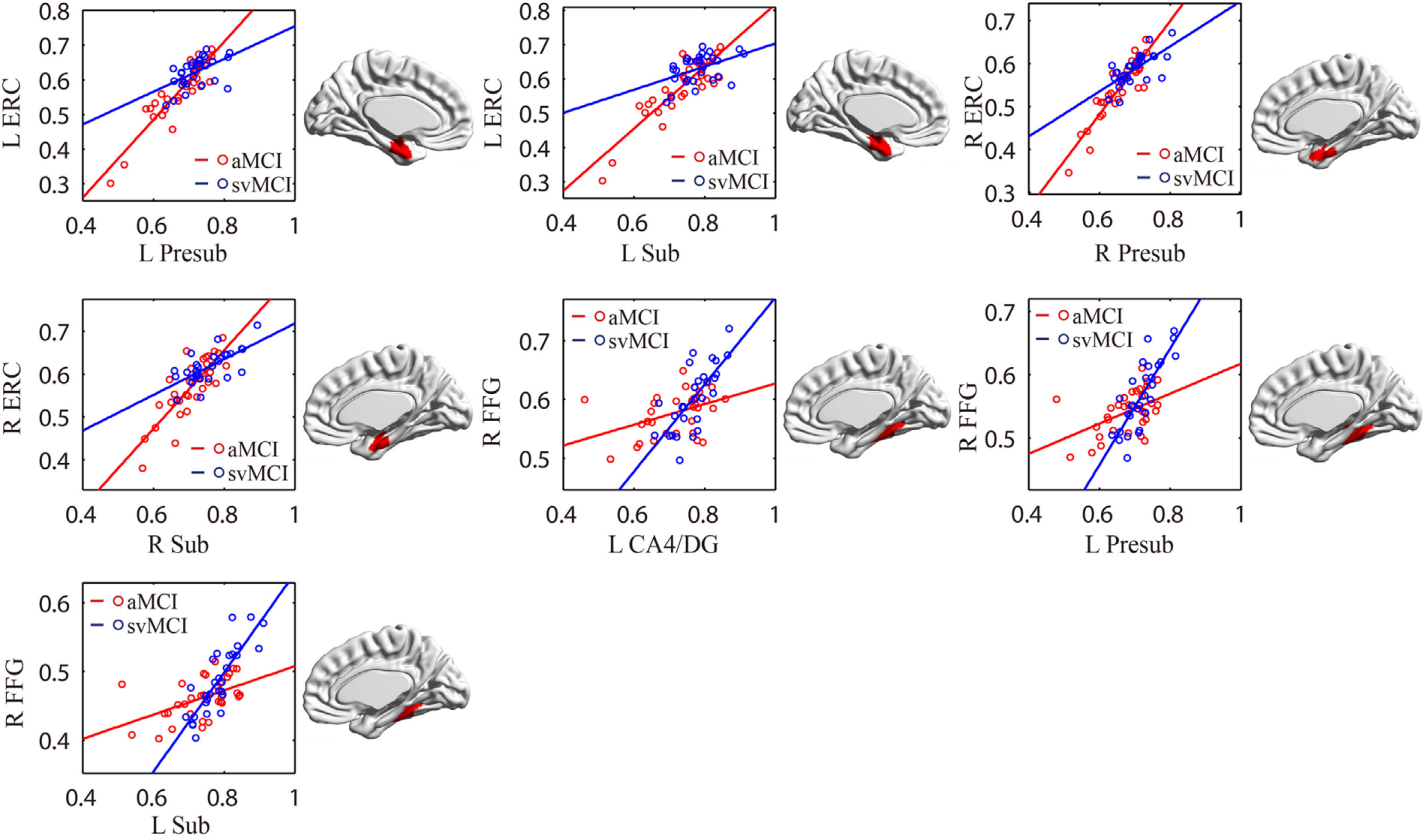
Significant between-group differences in structural association for aMCI and svMCI. A cluster showing significant structural difference (Gaussian random field-corrected at voxel level: *P* < 0.01 and cluster level: *P* < 0.05) is presented on the right, and a plot of slope differences between the seed region and cluster region is presented on the left. Abbreviations: L, left; R, right; aMCI, amnestic mild cognitive impairment; svMCI, subcortical vascular mild cognitive impairment; CA, cornu ammonis; DG, dentate gyrus; Presub, presubiculum; Sub, subiculum; ERC, entorhinal cortex; FFG, fusiform gyrus.

As shown in Table 2 and Figure 7, there were significant increased associations between several hippocampal subfields (bilateral presubiculum, bilateral subiculum) and the bilateral ERC in the aMCI group compared to the svMCI group. Then, left hippocampal subfields mostly showed decreased covariance with the right FFG in the aMCI group relative to the svMCI group.

## Discussion

In this study, we selected hippocampal subfields as seeds to build SCNs among three groups. Specifically, hippocampal subfields correlated with the TP, thalamus, subcallosal cortex, and posterior cingula cortex in the aMCI group, while hippocampal subfields significantly covaried with the OFC in the svMCI group. Finally, we compared the differences in strength of structural covariance between groups. The results demonstrated that there were abnormal structural associations between hippocampal subfields and the cerebral cortex in aMCI and svMCI patients, and these abnormalities were different between them.

### Structural Covariance Networks Within Groups

In our study, positive correlations between hippocampal subfields and FFG, and ERC in GM volume were found in each group. To some extent, these positive correlations suggested synchronous GM changes in these regions (29, 32). The ERC and FFG are anatomically adjacent to the hippocampus. Importantly, there were many intrinsic connections between the hippocampus, ERC, and FFG (7).

All the hippocampal subfields showed significantly positive structural covariance with the thalamus in the aMCI group. A previous study reported atrophy of the thalamus in aMCI patients (45). The positive structural covariance could be explained by the synchronous atrophy between the thalamus and hippocampus in the aMCI group. The subiculum and entorhinal cortices were found to project to the thalamus (1). There were many disruptions in the thalamus functional connectivity in aMCI including thalamo-hippocampus, thalamo-temporal, thalamo-visual, and thalamo-default network (46). Some cognitive impairments in aMCI, such as visual–spatial perception syndrome and visual hallucinations, may be due to thalamus atrophy and abnormalities in thalamus-related networks.

We also observed positive structural associations between the left hippocampal subfields and subcallosal cortex in the aMCI group. This suggested right subcallosal cortex atrophy in aMCI patients (47). In addition, significant correlations between cognitive scores on the episodic memory task and increased functional connectivity between the subcallosal cortex and hippocampus were found in aMCI patients (48). This indicated that the abnormal structural correlations in the subcallosal cortex could be related to the observed memory deficits in aMCI patients.

We found significantly positive structural associations between the right hippocampal subfields and PCC in the aMCI group. Many histopathological (49), structural (50), and functional imaging (51, 52) studies consistently reported that the PCC was an important structure in the pathophysiology of AD and aMCI. Importantly, the functional disconnection of hippocampal subregions and PCC may be a main factor of impaired episodic memory in aMCI (20). Because the developmental trajectory of the structural network may associate with its functional specialization (53), the abnormality between PCC and hippocampal subfields may underpin the episodic memory deficits observed in aMCI.

### Significant Differences in SCNs Between Groups

We observed that the increased connections between the right FFG and left presubiculum were stronger in svMCI than in the aMCI and NC groups. Previous studies have shown FFG atrophy in svMCI patients (24, 54). The FFG is related to semantic processing (55). Thus, the abnormal structural correlations between hippocampal subfields and the FFG could have an effect on the reduced capacity for semantic memory. Our results indicated that abnormality between the hippocampal subfields and FFG was distinct in svMCI, which was characterized by the main deficit of semantic memory compared to aMCI.

In the aMCI group, compared to the svMCI group, we observed an increased association between the bilateral presubiculum/subiculum and the ERC. The pathway from CA1 to the subiculum and projections to the ERC form the principal output from the hippocampus. The connections between CA1, subiculum, and ERC were associated with episodic memory processing (26). Therefore, the synchronous atrophy in the ERC and hippocampal subregions may suggest the disruption of episodic memory distinctly in aMCI patients.

The left CA1/CA4/DG/subiculum showed significantly increased structural association with the left TP in aMCI patients compared to NC. The stronger structural covariance potentially indicates synchronous GM changes in these regions affected by the disease (29). Thus, we speculate that the increased structural covariance between hippocampal subfields and the temporal gyrus suggests synchronous atrophy in the aMCI group. Several studies have shown atrophy in the temporal gyrus, especially in the medial and ITG, which supports our results (8, 56). The TP is associated with both social and emotional processes, which mainly involves face recognition and theory of mind (57). Chen et al. also indicated decreased connectivity between the middle hippocampus and middle temporal gyrus (MTG) in functional connectivity (26). We assumed that the synchronous atrophy between the hippocampus and MTG could explain the disrupted functional connectivity between them.

We also observed increased structural associations between the left CA1/subiculum and left POG in aMCI compared to NC. Left POG atrophy was reported in aMCI patients (58). Additionally, NC subjects had greater activations than aMCI patients during “Binds,” which probe object memory in the left POG, and our findings on the abnormal structural correlation with the left POG could be related to early signs of object memory deficits in aMCI patients (53).

In addition, bilateral CA1 and left CA2/3 showed significantly positive associations with the OFC in the svMCI group compared to the NC group. In addition, we found increased covariance between the right CA1 and right PFC in svMCI compared to NC. For PET imaging, the patients with svMCI showed hypometabolism in the inferior and medial frontal cortices adjacent to the cingulate gyrus (59). Importantly, the prefrontal gyrus is associated with executive function, which has a deficit in svMCI patients (60). Additionally, frontal-subcortical circuits, including hippocampus and OFC, mediate many aspects of human behavior, especially executive function (61). We assume that abnormal structural covariance between hippocampal subfields and the OFC indicates deficit of executive function in svMCI patients.

This study still has several limitations. First, we did not study the structural connectivity based on diffusion-weighted imaging (DWI) of the white matter pathway between hippocampal subfields and the whole brain cortex. There was a lack of systematic comparisons of structural covariance networks and DWI-based networks. Our findings of SCNs among the three groups should be cautiously extended to other structural networks. Second, in this study, due to the limited segmentation accuracies of hippocampal subfields in regular T1 images, we focused on the SCN analysis of each subfield instead of volumetric analysis of subfields. In the future, the volumetric analysis of hippocampal subfields would be helpful to understand the hippocampal abnormalities in the two MCI subtypes with the improvement of segmentation accuracies of hippocampal subfields in regular T1 images.

## Ethics Statement

This study was approved by the medical research ethics committee and the institutional review board of Xuanwu Hospital, Capital Medical University, Beijing, China.

## Author Contributions

Conceived and designed the experiments: SL and XL. Analyzed the data: XW. Contributed reagents/materials/analysis tools: YY, YH, CY, and WZ. Wrote the paper: XW, SL, XL, QL, and YY.

## Conflict of Interest Statement

The authors declare that the research was conducted in the absence of any commercial or financial relationships that could be construed as a potential conflict of interest.

## Footnotes

^1^http://freesurfer.net.

^2^http://www.fil.ion.ucl.ac.uk/spm.

^3^http://www.neuro.uni-jena.de/vbm/.

^4^http://www.nitrc.org/projects/bnv/.
